# Identification of individuals benefiting from the kakaritsuke-yakuzaishi (family pharmacist) system in Japan: a retrospective cohort study using an employment-based health insurance claims database

**DOI:** 10.1186/s12913-022-08093-0

**Published:** 2022-05-21

**Authors:** Ryo Iketani, Keiko Konomura

**Affiliations:** Center for Outcomes Research and Economic Evaluation for Health, National Institute of Public Health, 2-3-6 Minami, Wako, Saitama 351-0197 Japan

**Keywords:** Additive interaction, Administrative claims data, Community pharmacy, Pharmacist, Retrospective cohort study

## Abstract

**Background:**

The kakaritsuke-yakuzaishi system (henceforth, the family pharmacist system) which provides more health services than those by general pharmaceutical practice, was implemented in Japan in April 2016. To distribute medical resources and medical care expenditures appropriately, identifying the possible major beneficiaries of this system is essential. By analyzing administrative claims data through this retrospective cohort study, we identified modifiers of the potential benefits of the system. Further, we integrated the identified modifiers into a scoring system that indicates the possible benefitting subpopulations.

**Methods:**

We obtained data about individuals under 75 years old routinely using community pharmacies in Japan from the JMDC database. We classified the individuals as users or non-users. We used claims related to “choufukutouyaku-sougosayoutou-boushi-kasan (additional therapeutic duplication and drug interaction [TDDI] prevention fees)” filed between April 2018 and March 2020, which indicate that individuals’ prescriptions were modified to adjust leftover drugs or to avoid TDDI as indicators of potential benefit. We estimated adjusted absolute risk differences and 95% confidence intervals for product terms using multiple generalized linear regression models. We included the factors whose 95% confidence interval lower limits did not reach 0 in the multiple logistic regression models for developing a scoring system.

**Results:**

The eligible cohort included 162,340 individuals (1,214 users and 161,126 non-users). The leftover drugs adjustment significantly increased for individuals prescribed antidepressants. However, as only one modifier was identified, we did not develop a scoring system for the leftover drugs adjustment. For TDDI prevention, the following factors were included in the scoring system: being female, being prescribed ≥ 6 drug types, using ≥ 2 medical institutions, and being prescribed proton pump inhibitors, antibiotics, probiotics, or traditional Japanese herbal medicines. The developed scoring system for TDDI prevention scored “female” and “traditional Japanese herbal medicines prescription” factors higher than other factors.

**Conclusions:**

Individuals who are female or prescribed traditional Japanese herbal medicines, or antidepressants may benefit significantly from the family pharmacist system.

**Supplementary Information:**

The online version contains supplementary material available at 10.1186/s12913-022-08093-0.

## Introduction

Japan’s universal health care system reimburses various health services, including those provided in community pharmacies, in the country [1–3]. From drug dispensing fee to individuals’ pharmacotherapies management fee reimbursements, compensation amounts are carefully set in health care plans. These plans are reviewed biennially, allowing for the addition of new health services coverage or compensation amount modifications [3]. This review is done, in part, to facilitate certain health services. For example, the separation between prescribing and dispensing is facilitated by developing a system through which physicians receive higher fees for fulfilling prescriptions to community pharmacies, compared with the fees they receive for dispensing drugs themselves [4]. However, this system’s expectations have not been fully met, as community pharmacies in Japan are usually located in front of hospitals. Therefore, individuals often visit different pharmacies depending on the hospital from which their physicians prescribe drugs [5]. Thus, it has been difficult to provide centralized and consistent pharmaceutical care in Japan.

In April 2016, the kakaritsuke-yakuzaishi system (henceforth, the family pharmacist system) was implemented in Japan by adjusting the kakaritsuke-yakuzaishi-shidouryo (henceforth, the family pharmacist consultation fees) to assist community pharmacies. The family pharmacist system aims to facilitate centralized and consistent pharmaceutical care [5, 6] and enable the delivery of more effective and comprehensive pharmaceutical services by developing patient–pharmacist contracts. To claim fees within this system, family pharmacists must provide various services, such as 24-h availability for consultations, tracking dispensing information from other pharmacies, and gathering feedback from physicians. Although these services may form part of general pharmaceutical practice, this system differs in that these services are clearly defined as conditions to claim the established fees. In exchange for these additional services, family pharmacists are incentivized by a higher fee than that obtained in general practice.

Since its inception, this system has proven to be successful. One study suggested that the family pharmacist system facilitated therapeutic duplication and drug interaction (TDDI) prevention and leftover drugs adjustment [7]. Another study corroborated these findings, stating that paying higher fees to pharmacists providing comprehensive pharmaceutical services facilitated pharmaceutical management [8]. However, it remains unclear what kind of individuals benefit the most from this system. Identifying the system’s target population and the population for whom general pharmaceutical care is sufficient is crucial to ensure the appropriate medical resources delivery and medical care expenditures.

Therefore, through this retrospective cohort study, we identified the factors that modified the benefit that individuals obtained from the family pharmacist system by analyzing administrative claims data. We also attempted to develop a scoring system using the identified modifiers to investigate subpopulations that can benefit the most through this system.

## Methods

### Data source and cohort definition

We sourced data from the JMDC database (JMDC Inc., Tokyo, Japan), which contains basic information (e.g., month and year of birth, sex, and enrollment period in the database) and administrative claims data collected from 11,065,484 individuals under 75 years old (as of January 2021). Claims data includes diagnoses based on International Classification of Disease (10th revision) codes and prescribed drugs based on the WHO’s Anatomical Therapeutic Chemical classification system codes. All data are anonymized and linked by unique identifiers. The details of this database are presented in previous reports [9, 10].

We obtained data from 1.7 million individuals randomly sampled from the database. To account for the biennial health care plan reviews, we set the observational period between April 2018 and March 2020, and the pre-observational period between April 2017 and March 2018.

To identify the eligible cohort, first, we selected individuals who used the same community pharmacy at least once every six months during the pre-observational period from the database. Then, we divided individuals into users and non-users of the family pharmacist system based on the family pharmacist consultation fee claims during the pre-observational period. Lastly, we excluded individuals who met the following exclusion criteria from the eligible cohort: (1) being under 18 years old at the start of the observational period; (2) not receiving any pharmaceutical management during the observational period; (3) switching groups (user/non-user) during the observational period; (4) being unable to claim the fee related to the endpoint; (5) receiving pharmaceutical management at home or at a nursing home; and (6) being unable to be observed until March 31, 2020.

### Endpoint

We used “choufukutouyaku-sougosayoutou-boushi-kasan” (henceforth, additional TDDI prevention fee) claims as indicators of potential benefit from pharmaceutical management. This additional TDDI prevention fee is divided into two types of claims, one related to leftover drugs adjustment and the other to TDDI prevention. We evaluated each claim separately to identify the modifiers of benefits resulting from the family pharmacist system.

### Candidate variables

The variables used in the analyses and their definitions are shown in Table 1. We analyzed these variables as potential benefit modifiers, except for “number of medical examinations” and “use of a pharmacy that can claim the family pharmacist consultation fees,” which we used as confounding factors instead to deal with potential heterogeneity in the frequency and quality of pharmaceutical services. We categorized age, types of drugs, and the number of medical institutions used based on a previous study [7], and collected data (excluding data on age and sex) six months prior to the observational period.

**Table 1:** The definitions of variables

Variables	Definitions
Age	age at the beginning of the observation period (April 1, 2018)
Sex	male/female
Types of drugs	the sum of drugs in different chemical subgroups based on WHO-ATC codes
Medical institutions used	the sum of visits to medical institutions
Use of multiple departments in a hospital	the claims of fees for using multiple departments in a hospital
The number of medical examinations	the sum of different days when individuals visited medical institutions and were prescribed drugs
Admission	the record of either admission or the claims of the fees for admission
Use of a pharmacy that can claim the family pharmacist consultation fees	the use of a pharmacy that claimed the family pharmacist consultation fees
Use of one-dose package for drugs	the claim of the fee for packaging drugs in one dose
Drugs	
Antihypertensives	WHO-ATC code: C02-, C03-, C07-, C08-, C09-, C11-, C10BX03, C10BX04, C10BX06, C10BX07, C10BX09, C10BX10, C10BX11, C10BX12, C10BX13, C10BX14, C10BX15, C10BX16 or C10BX17
Antilipidemic agents	WHO-ATC code: C10-
Antidiabetic agents other than insulin	WHO-ATC code: A10M-, A10BA-, A10BB-, A10BD-, A10BF-, A10BG-, A10BH-, A10BJ- or A10BK-
Insulin	WHO-ATC code: A10A-
Anticoagulants	WHO-ATC code: B01AA03, B01AE07, B01AF01, B01AF02, B01AF03 or B01AF04 in oral formulation
Antiplatelet agents	WHO-ATC code: B01AC04, B01AC05, B01AC06, B01AC24, B01AC25, B01AC30, B01AC56, A02BC53 or A02BC54 in oral formulation
Proton pump inhibitors	WHO-ATC code: A02BC-, A02BD-, B01AC56, A02BC53 or A02BC54 in oral formulation
H2 blockers	WHO-ATC code: A02BA- in oral formulation
H1 blockers	WHO-ATC code: R06A- in oral formulation
Antipsychotics	WHO-ATC code: N05A- in oral formulation
BZDs/Non-BZDs	WHO-ATC code: N05BA-, N05CD- or N05CF- in oral formulation
Antidepressants	WHO-ATC code: N06A- in oral formulation
Antibiotics	WHO-ATC code: J01- in oral formulation
NSAIDs	WHO-ATC code: M01A-, M02AA-, M02AC-, M02BG-, N02AE01, N02AJ13, N02AX02, N02BE71, or N02CA52 in oral formulation or external preparation
Steroids	WHO-ATC code: A23A- in oral formulation or external preparation
Probiotics	WHO-ATC code: A07F-
Laxatives	WHO-ATC code: A06-
Vitamins	WHO-ATC code: A11-, B03BA- or B02BA- in oral formulation
Traditional Japanese herbal medicines	WHO-ATC code: V03B1-

*Abbreviations:* *BZDs* Benzodiazepines, *NA* Not Applicable, *NSAIDs* Non-steroidal Anti-inflammatory Drugs, *WHO-ATC* The World Health Organization’s Anatomical Therapeutic Chemical Classification System

Data (excluding age and sex) were collected for a period of six months before April 1, 2018

### Statistical analysis

We summarized continuous variables and categorical variables as means with standard deviations and frequencies (%), respectively, and calculated the proportion of the additional TDDI prevention fee claim.

We applied multiple generalized linear regression model (link: identity; distribution: binomial) including the variables listed in Table 1 to estimate the adjusted absolute risk differences (aARDs) and 95% confidence intervals (CIs) for the association between the use of the family pharmacist system and the additional TDDI prevention fee claim [11]. To identify the modifiers of benefit, we developed models that include the use of the family pharmacist system, one candidate variable, a product term between the use of the system and one candidate variable, and other variables listed in Table 1 [12]. If the product terms’ 95% CI lower limits did not reach 0, we extracted the variables as the modifiers. We implemented sensitivity analyses that included individuals who switched groups (user/non-user) during the observational period in the eligible cohort to account for any differences in the probability that they claimed the additional TDDI prevention fee.

Based on the identified modifiers, we developed two scoring systems to identify subpopulations for which the benefit of using the system was enhanced, using the method shown by Zhao et al. [13, 14]. First, we divided the eligible cohort randomly into the training dataset (75%) and the test dataset (25%). Second, we conducted the following two modeling procedures for the training dataset: Modeling Procedure I, wherein we applied two multiple logistic regression models separately to each group (users/non-users) and Modeling Procedure II, wherein we applied a single multiple logistic regression model. Then, we created two separate models, collectively named Model I, composed of the identified modifiers and other variables listed in Table 1, and created a single model, referred to as Model II, comprising the use of the system, identified modifiers, product terms between the use of the system and the identified modifiers, and other variables listed in Table 1. We then applied Modeling Procedures I and II with stepwise selection, wherein we selected and eliminated terms that reached the 0.20 level of significance. The results are presented in Additional file 1. Third, we estimated the scores using the differences in Model I’s coefficients and using Model II’s coefficients of the product terms. Fourth, we calculated the distributions of the scores for the training dataset and the aARDs in each subpopulation having equal or higher q-th percentiles. The higher the aARDs were, the greater was the potential benefit for the analyzed subpopulation. We plotted graphs with q in the x-axis and corresponding aARDs in the y-axis, and calculated the area under the aARD curves (AUCs). Fifth, we applied the methodology described in the fourth step to the test dataset using the scoring systems developed in the second and third steps. Lastly, we compared the plots and aARDs obtained from the training dataset and test dataset to evaluate overoptimism. We determined the best scoring system based on higher AUC from the training dataset.

We conducted all statistical analyses using SAS software, version 9.4 for Windows (SAS institute Inc., Cary, NC, USA).

## Results

### Eligible cohort

**Fig. 1:**
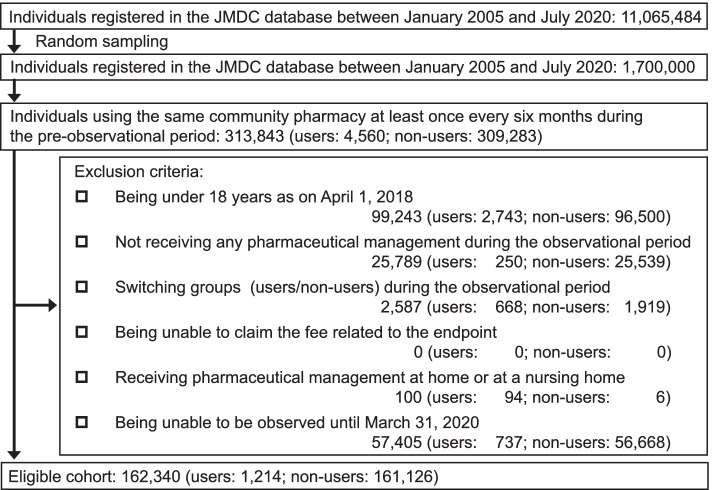
Flow diagram of identifying the eligible cohort

Among the 1.7 million individuals in the JMDC database, 162,340 met this study’s eligibility criteria (users: 1,214; non-users: 161,126) (Fig. 1). Table 2 shows their characteristics. We observed claims related to the adjustment of leftover drugs in 6.9% (84) of the user group and 2.7% (4,318) of the non-user group (aARD: 1.9; 95% CI: 0.6 to 3.2). We observed TDDI prevention claims in 8.9% (108) of the user group and 3.2% (5,168) of the non-user group (aARD, 1.9; 95% CI, 0.3 to 3.4).

**Table 2:** Background characteristics

Variables	User (*N* = 1214)	Non-user (*N* = 161,126)
Age, years	50.2 (12.0)	46.6 (12.1)
≥ 60 years	286 (23.6)	22,805 (14.2)
Sex (Female)	583 (48.0)	74,899 (46.5)
Types of drugs	10.5 (7.0)	8.5 (5.8)
≥ 6	900 (74.1)	102,544 (63.6)
Medical institutions used	2.3 (1.3)	2.1 (1.2)
≥ 2	816 (67.2)	103,253 (64.1)
Use of multiple departments in a hospital	109 (9.0)	5808 (3.6)
The number of medical examinations	9.7 (10.9)	6.0 (5.5)
Admission	69 (5.7)	4789 (3.0)
Use of a pharmacy that can claim the family pharmacist consultation fees	1214 (100.0)	35,350 (21.9)
Use of one dose package for drugs	84 (6.9)	2159 (1.3)
Drugs		
Antihypertensives	462 (38.1)	38,293 (23.8)
Antilipidemic agents	348 (28.7)	27,918 (17.3)
Antidiabetic agents other than insulin	147 (12.1)	11,495 (7.1)
Insulin	30 (2.5)	1904 (1.2)
Anticoagulants	34 (2.8)	1639 (1.0)
Antiplatelet agents	63 (5.2)	4076 (2.5)
Proton pump inhibitors	220 (18.1)	16,161 (10.0)
H2 blockers	103 (8.5)	10,007 (6.2)
H1 blockers	422 (34.8)	59,920 (37.2)
Antipsychotics	138 (11.4)	6471 (4.0)
BZDs/Non-BZDs	297 (24.5)	20,260 (12.6)
Antidepressants	162 (13.3)	9900 (6.1)
Antibiotics	443 (36.5)	63,446 (39.4)
NSAIDs	497 (40.9)	60,864 (37.8)
Steroids	138 (11.4)	12,450 (7.7)
Probiotics	191 (15.7)	20,693 (12.8)
Laxatives	91 (7.5)	7052 (4.4)
Vitamins	149 (12.3)	14,829 (9.2)
Traditional Japanese herbal medicines	282 (23.2)	30,941 (19.2)

*Abbreviations:* *BZDs* Benzodiazepine, *NSAIDs* Non-steroidal Anti-inflammatory Drugs

Continuous variables and categorical variables are summarized as means with standard deviations and frequencies (%), respectively

### Identification of modifiers

Regarding the leftover drugs adjustment claims, we identified the prescription of antidepressants as a modifier (Table 3). The sensitivity analysis indicates similar results (see eTable 1 in Additional file 1).

**Table 3:** The evaluation of additive interaction for the adjustment of leftover drugs

Variables	Levels	Non-user	User	aARDs of product terms (95% CIs)
Age	< 60 years	REF	2.2	-1.5 (-4.8 to 1.8)
	≥ 60 years	1.1	1.7	
Sex	Male	REF	2.5	-1.2 (-3.8 to 1.5)
	Female	0.1	1.4	
Types of drugs	< 6	REF	2.1	-0.2 (-3.1 to 2.6)
	≥ 6	0.3	2.1	
Medical institutions used	< 2	REF	2.3	-0.6 (-3.4 to 2.1)
	≥ 2	-0.2	1.4	
Use of multiple departments in a hospital	No	REF	1.9	0.6 (-5.0 to 6.1)
	Yes	1.0	3.4	
Admission	No	REF	1.7	6.3 (-2.3 to 14.9)
	Yes	0.3	8.3	
Use of one-dose package for drugs	No	REF	1.8	3.8 (-3.9 to 11.6)
	Yes	3.2	8.8	
Antihypertensives	No	REF	1.6	1.0 (-2.0 to 3.9)
	Yes	1.0	3.5	
Antilipidemic agents	No	REF	2.5	-2.0 (-5.2 to 1.2)
	Yes	1.2	1.6	
Antidiabetic agents other than insulin	No	REF	1.9	-0.7 (-5.8 to 4.5)
	Yes	3.8	5.1	
Insulin	No	REF	1.7	14.3 (-1.6 to 30.2)
	Yes	1.6	17.6	
Anticoagulants	No	REF	1.9	-0.4 (-9.8 to 9.0)
	Yes	2.1	3.5	
Antiplatelet agents	No	REF	2.0	-2.0 (-8.7 to 4.7)
	Yes	1.7	1.6	
Proton pump inhibitors	No	REF	1.7	1.7 (-2.5 to 5.8)
	Yes	1.2	4.6	
H2 blockers	No	REF	2.0	-1.7 (-6.5 to 3.1)
	Yes	0.3	0.6	
H1 blockers	No	REF	1.7	0.4 (-2.4 to 3.2)
	Yes	0.1	2.3	
Antipsychotics	No	REF	1.6	3.5 (-1.8 to 8.9)
	Yes	0.8	5.9	
BZDs/Non-BZDs	No	REF	1.4	2.3 (-1.2 to 5.8)
	Yes	0.5	4.2	
Antidepressants	No	REF	1.3	5.2 (0.01 to 10.3)
	Yes	0.5	7.0	
Antibiotics	No	REF	2.1	-0.5 (-3.3 to 2.2)
	Yes	-0.1	1.5	
NSAIDs	No	REF	2.5	-1.8 (-4.5 to 0.9)
	Yes	0.1	0.8	
Steroids	No	REF	2.0	-1.0 (-5.2 to 3.2)
	Yes	0.4	1.4	
Probiotics	No	REF	1.8	0.5 (-3.3 to 4.4)
	Yes	0.1	2.4	
Laxatives	No	REF	1.9	0.3 (-5.7 to 6.2)
	Yes	0.4	2.5	
Vitamins	No	REF	1.9	0.0 (-4.3 to 4.4)
	Yes	0.2	2.2	
Traditional Japanese herbal medicines	No	REF	2.0	-0.4 (-3.6 to 2.7)
	Yes	0.3	1.9	

*Abbreviations:* *aARDs* adjusted Absolute Risk Differences, *BZDs* Benzodiazepine, *CIs* Confidence Intervals, *NSAIDs* Non-steroidal Anti-inflammatory Drugs, *REF* Reference

Value in each stratum indicates aARDs from a common REF stratum

Regarding TDDI prevention claims, we identified being female, being prescribed ≥ 6 types of drugs, using ≥ 2 medical institutions, and being prescribed proton pump inhibitors, antibiotics, probiotics, or traditional Japanese herbal medicine as modifiers (Table 4). The sensitivity analysis indicates similar results (see eTable 2 in Additional file 1).

**Table 4:** The evaluation of additive interaction for the prevention of therapeutic duplication or drug interaction

Variables	Levels	Non-user	User	aARDs of product terms (95% CIs)
Age	< 60 years	REF	2.1	-0.8 (-4.4 to 2.8)
	≥ 60 years	0.5	1.8	
Sex	Male	REF	-0.2	4.7 (1.6 to 7.9)
	Female	0.5	5.1	
Types of drugs	< 6	REF	-1.0	4.4 (1.7 to 7.1)
	≥ 6	0.0	3.4	
Medical institutions used	< 2	REF	-0.9	4.5 (1.8 to 7.3)
	≥ 2	-0.1	3.5	
Use of multiple departments in a hospital	No	REF	1.4	7.0 (-0.5 to 14.5)
	Yes	2.2	10.6	
Admission	No	REF	1.7	4.1 (-4.2 to 12.4)
	Yes	0.5	6.2	
Use of one-dose package for drugs	No	REF	1.6	5.9 (-2.4 to 14.1)
	Yes	1.8	9.2	
Antihypertensives	No	REF	1.7	0.5 (-2.8 to 3.7)
	Yes	0.0	2.2	
Antilipidemic agents	No	REF	2.2	-1.3 (-4.6 to 2.1)
	Yes	0.4	1.4	
Antidiabetic agents other than insulin	No	REF	1.6	2.6 (-2.7 to 7.9)
	Yes	0.6	4.7	
Insulin	No	REF	1.6	14.6 (-1.6 to 30.8)
	Yes	1.1	17.3	
Anticoagulants	No	REF	1.7	6.0 (-6.9 to 18.9)
	Yes	0.5	8.2	
Antiplatelet agents	No	REF	1.9	0.4 (-7.4 to 8.1)
	Yes	-0.3	2.0	
Proton pump inhibitors	No	REF	1.1	5.8 (0.7 to 10.8)
	Yes	1.7	8.6	
H2 blockers	No	REF	1.8	0.7 (-5.7 to 7.0)
	Yes	1.4	3.9	
H1 blockers	No	REF	0.9	3.1 (-0.3 to 6.6)
	Yes	0.7	4.7	
Antipsychotics	No	REF	1.6	2.3 (-3.1 to 7.7)
	Yes	0.4	4.3	
BZDs/Non-BZDs	No	REF	1.1	3.6 (-0.5 to 7.7)
	Yes	0.5	5.2	
Antidepressants	No	REF	2.0	-0.9 (-5.4 to 3.7)
	Yes	0.0	1.1	
Antibiotics	No	REF	0.0	5.5 (2.0 to 9.0)
	Yes	0.0	5.6	
NSAIDs	No	REF	1.5	1.0 (-2.2 to 4.3)
	Yes	0.3	2.8	
Steroids	No	REF	1.4	5.0 (-1.2 to 11.1)
	Yes	0.8	7.1	
Probiotics	No	REF	0.8	8.0 (2.4 to 13.5)
	Yes	0.3	9.1	
Laxatives	No	REF	1.7	3.6 (-4.2 to 11.3)
	Yes	0.4	5.7	
Vitamins	No	REF	1.7	2.4 (-3.4 to 8.1)
	Yes	0.7	4.7	
Traditional Japanese herbal medicines	No	REF	0.5	7.0 (2.5 to 11.5)
	Yes	0.5	8.0	

*Abbreviations:* *aARDs* adjusted Absolute Risk Differences, *BZDs* Benzodiazepine, *CIs* Confidence Intervals, *NSAIDs* Non-steroidal Anti-inflammatory Drugs, *REF* Reference

Value in each stratum indicates aARDs from a common REF stratum

### Scoring

We divided the cohort into 121,755 individuals in the training dataset (users: 901; non-users: 120,854) and 40,585 individuals in the test dataset (users: 313; non-users: 40,272).

**Fig. 2:**
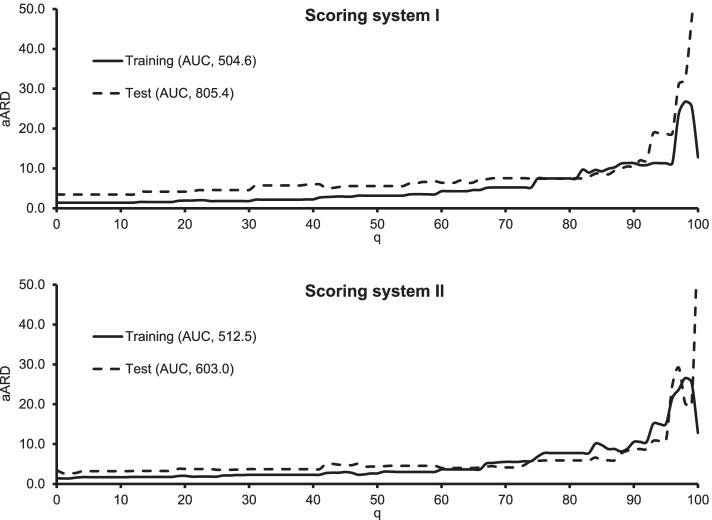
Adjusted absolute risk difference curves for the prevention of therapeutic duplication or drug interaction. Abbreviations*.* aARD: adjusted absolute risk difference; AUC: area under the absolute risk difference curve. Scoring system I was developed using two multiple logistic regression models and scoring system II was developed using a single multiple logistic regression model. The plots depict q in the x-axis and the corresponding aARDs in the y-axis based on these scoring systems. The higher the AUC is, the better the scoring system performs

We did not develop a scoring system for leftover drugs adjustment claims, as, except for antidepressant prescription, there were no variables to be considered. Conversely, we developed two scoring systems for TDDI prevention claims from the training dataset (Table 5). The coefficients from the multiple logistic regression model are shown in eTable 3 (see Additional file 1). Table 6 indicates the estimated aARDs in subpopulations having an equal or higher q-th percentile scores from both the training and test datasets. Figure 2 depicts the correspondences between q and aARDs. Scoring system II indicated higher AUC (512.5).

**Table 5:** Score allocations of the identified modifiers for the prevention of therapeutic duplication or drug interaction

Variables	Scoring system I	Scoring system II
Sex (Female)	0.58	0.49
Types of drugs (≥ 6)	0.16	-0.05
Medical institutions used (≥ 2)	0.08	-0.07
Proton pump inhibitors	0.08	-0.17
Antibiotics	0.01	-0.03
Probiotics	0.32	0.29
Traditional Japanese herbal medicines	0.37	0.45

**Table 6:** Correspondence between scores and aARDs for the prevention of therapeutic duplication or drug interaction

**q**	**Scoring system I**				**Scoring system II**			
	**Training**		**Test**		**Training**		**Test**	
	**Scores**	**aARDs**	**Scores**	**aARDs**	**Scores**	**aARDs**	**Scores**	**aARDs**
0	0.00	1.4	0.00	3.5	-0.32	1.4	-0.32	3.5
10	0.00	1.4	0.00	3.5	-0.15	1.7	-0.15	3.2
20	0.16	1.9	0.16	4.2	-0.08	2.0	-0.08	3.8
30	0.24	1.8	0.24	4.6	0.00	2.3	0.00	3.7
40	0.32	2.2	0.32	5.9	0.00	2.3	0.00	3.7
50	0.58	3.2	0.58	5.6	0.30	2.6	0.30	4.4
60	0.66	4.3	0.66	6.4	0.37	3.6	0.37	4.0
70	0.82	5.2	0.82	7.6	0.42	5.5	0.42	4.2
80	0.83	7.5	0.83	7.4	0.49	7.8	0.49	5.9
90	1.15	11.3	1.15	10.5	0.79	10.6	0.79	8.8
100	1.60	12.8	1.60	64.2	1.23	12.8	1.23	64.2
AUCs	504.6		805.4		512.5		603.0	

*Abbreviations:* *aARDs* adjusted Absolute Risk Differences, *AUCs* the Areas Under the adjusted absolute risk difference Curves

## Discussion

Here, we identified modifiers of benefitting from the family pharmacist system in Japan. We identified the prescription of antidepressants as a modifier of leftover drugs adjustment claims. The family pharmacist system is possibly more effective for those who were prescribed antidepressants because low adherence rates to them are often problematic [15]. Moreover, seven factors—being female, being prescribed ≥ 6 types of drugs, using ≥ 2 medical institutions, and being prescribed proton pump inhibitors, antibiotics, probiotics, or traditional Japanese herbal medicines, were identified as modifiers of TDDI prevention claims. This suggests that TDDI prevention through the family pharmacist system was associated with those factors.

For TDDI prevention claims, the scoring systems used to detect subpopulations for which benefit was enhanced were developed using two distinct procedures based on the seven identified factors. Each scoring system depicted similar aARD curves and values between the training and the test datasets. These systems indicated that the aARD increased as the score increased, and the benefit obtained from the system was enhanced in subpopulations with scores over 70–80 percentiles. Individuals with scores over 70–80 percentiles corresponded to females prescribed probiotics or traditional Japanese herbal medicines in both scoring systems. Further, all scoring systems including systems with stepwise selection included being female and being prescribed traditional Japanese herbal medicine and allocated higher scores to them. Therefore, individuals having these factors may benefit significantly from the system. When applied to clinical practice, scoring system II seems to have performed better, as it indicated higher AUC in the training dataset. Scoring system I seemed useful as well, as it indicated similar AUC as system II.

Among the factors identified as modifiers for TDDI prevention claims, the use of multiple medical institutions would be consistent with the factor that the system expected in individuals [5]. As mentioned earlier, the objective of the family pharmacist system is to centralize pharmaceutical management. The prescription of ≥ 6 types of drugs is often recognized as a status requiring pharmaceutical attention or may reflect patient morbidity in the present study. However, the scoring systems allocated low scores to the use of multiple medical institutions and prescription of ≥ 6 types of drugs. These results suggested that the use of multiple medical institutions and prescription of ≥ 6 types of drugs essentially reflected the status of other identified modifiers indicating higher scores, specifically, being female and being prescribed traditional Japanese herbal medicines. As mentioned above, those who were female or prescribed traditional Japanese herbal medicines seemed to benefit significantly from the family pharmacist system.

An analysis of gender differences showed that the benefits may vary according to individuals’ health concerns, differences in medication intake, and the distribution of diseases requiring pharmaceutical treatment. Additionally, drug use during pregnancy is a major concern for women. It will be necessary for future research to interpret these results carefully and investigate the mechanism underlying the modifiers analyzed here.

Each drug identified as a modifier for TDDI prevention claims often results in typical prescription questions in Japan [16]. Thus, although the system may be useful in preventing TDDI in individuals who were prescribed these drugs, particularly traditional Japanese herbal medicines, these results need to be interpreted considering the following limitations.

First, we were unable to clarify whether these drugs truly caused TDDI, as data on prescribed drugs were obtained in the pre-observational period. Additionally, data on changes to inadequate prescriptions could not be retrieved due to data unavailability. We might not identify the modifiers but can ascertain the characteristics of patients experiencing TDDI and leftover drugs. Second, analyzing administrative claims data accumulated under the universal health care system in Japan allowed this study to evaluate the benefit of the services provided by the family pharmacist system; however, while confounding by indication was an issue, the impact may not be fully removed. For example, the use of over-the-counter drugs, treatment adherence, and individuals’ health and medicine concerns could potentially affect the association between the use of the system and the claim of additional TDDI prevention fees. This implied that the risks of experiencing TDDI and leftover drugs were still higher in the user group than those in non-user group after adjusting observable confounding factors. Identifying the modifiers of benefit requires controlling the confounding factors between the modified factor and the outcomes [12, 14]. Thus, the present study could not strictly identify the modifiers but could detect associations merely due to the first and second limitations.

The third limitation is related to the study’s generalizability. The JMDC database used in the present study does not contain sufficient data on the elderly. Therefore, the results may only be applicable to a younger population, compared with the one targeted by the system. The variable’s degree of modification may vary in populations including the elderly because the risk of experiencing drug-related problems increases with age [17–19]. Therefore, generalizing these results to the elderly should be done with caution. Other new factors and increases in modification using the identified factors may be detected by including the elderly.

These limitations caused by data availability made it difficult to infer whether the identified factors modified the benefit of the family pharmacist system for patients visiting pharmacies in Japan. Future studies using actual patient medication records in pharmacies can overcome these limitations. However, to explore multiple modifiers, a large sample size and cooperation of pharmacies or long study duration will be necessary because the frequency of claiming additional TDDI prevention fees was approximately 5%. Thus, at first, it is expected that future studies using actual patient medication records in pharmacies will focus on elucidating the mechanisms of the associations observed in this study.

## Conclusions

In this study, we identified the prescription of antidepressants as a modifier between the use of the family pharmacist system and leftover drugs adjustment. Additionally, we identified seven modifiers of the association between the use of the system and TDDI prevention. Of these seven modifiers, being female and being prescribed traditional Japanese herbal medicines may be most beneficial. Moreover, we developed scoring systems to identify which subpopulations benefited the most from the system using the modifiers for TDDI prevention claims. These scoring systems proved useful in assessing whether individuals benefited from the family pharmacist system.

## Supplementary Information

**Additional file 1:** **eTable 1.** The sensitivity analysis of additive interaction for the adjustment of leftover drugs (including individuals who switched groups). **eTable 2.** The sensitivity analysis of additive interaction for the prevention of therapeutic duplication or drug interaction (including individuals who switched groups). **eTable 3.** The coefficients of multiple logistic regression models for the scoring system I and II for the prevention of therapeutic duplication or drug interaction. **eTable 4.**The coefficients of multiple logistic regression models for the scoring system I and II for the prevention of therapeutic duplication or drug interaction with stepwise selection. **eTable 5. **Score allocations of the identified modifiers for the prevention of therapeutic duplication or drug interaction from scoring system I and II with stepwise selection. **eTable 6. **Correspondence between scores and aARDs for the prevention of therapeutic duplication or drug interaction from scoring system I and II with stepwise selection. **eFigure 1.** Adjusted absolute risk difference curves for the prevention of therapeutic duplication or drug interaction from scoring system I and II with stepwise selection.

## Data Availability

The data that support the findings of this study are available from JMDC Inc., but restrictions apply to the availability of these data, which were used under license for the current study and are not publicly available. Data are, however, available from the authors upon request and with permission of JMDC Inc. In that case, the corresponding author should be contacted.

## Abbreviations

aARDs: Adjusted absolute risk differences
AUCs: The areas under the adjusted absolute risk difference curves
BZDs: Benzodiazepines
CIs: Confidence intervals
NSAIDs: Non-steroidal anti-inflammatory drugs
NA: Not applicable
REF: Reference
TDDI: Therapeutic duplication and drug interaction
WHO-ATC: The World Health Organization’s Anatomical Therapeutic Chemical Classification System
