# Supplementary material for: Identification of individuals benefiting from the kakaritsuke-yakuzaishi (family pharmacist) system in Japan: a retrospective cohort study using an employment-based health insurance claims database
Source: BMC Health Serv Res. 2022 May 21;22:682. doi: 10.1186/s12913-022-08093-0 (PMC9124427; doi:10.1186/s12913-022-08093-0)
Supplement: Supplementary file 1 — Additional file 1: eTable 1. The sensitivity analysis of additive interaction for the adjustment of leftover drugs (including individuals who switched groups). eTable 2. The sensitivity analysis of additive interaction for the prevention of therapeutic duplication or drug interaction (including individuals who switched groups). eTable 3. The coefficients of multiple logistic regression models for the scoring system I and II for the prevention of therapeutic duplication or drug interaction. eTable 4.The coefficients of multiple logistic regression models for the scoring system I and II for the prevention of therapeutic duplication or drug interaction with stepwise selection. eTable 5. Score allocations of the identified modifiers for the prevention of therapeutic duplication or drug interaction from scoring system I and II with stepwise selection. eTable 6. Correspondence between scores and aARDs for the prevention of therapeutic duplication or drug interaction from scoring system I and II with stepwise selection. eFigure 1. Adjusted absolute risk difference curves for the prevention of therapeutic duplication or drug interaction from scoring system I and II with stepwise selection. [file 12913_2022_8093_MOESM1_ESM.docx]

**Additional file 1**

**Title:**

Identification of individuals benefiting from the kakaritsuke-yakuzaishi (family pharmacist) system in Japan: A retrospective cohort study using an employment-based health insurance claims database

**Authors:**

Ryo Iketani and Keiko Konomura.

**Contents**

**eTable 1**. The sensitivity analysis of additive interaction for the adjustment of leftover drugs (including individuals who switched groups).

**eTable 2**. The sensitivity analysis of additive interaction for the prevention of therapeutic duplication or drug interaction (including individuals who switched groups).

**eTable 3.** The coefficients of multiple logistic regression models for the scoring system I and II for the prevention of therapeutic duplication or drug interaction.

**eTable 4.** The coefficients of multiple logistic regression models for the scoring system I and II for the prevention of therapeutic duplication or drug interaction with stepwise selection.

**eTable 5.** Score allocations of the identified modifiers for the prevention of therapeutic duplication or drug interaction from scoring system I and II with stepwise selection.

**eTable 6.** Correspondence between scores and aARDs for the prevention of therapeutic duplication or drug interaction from scoring system I and II with stepwise selection.

**eFigure 1**. Adjusted absolute risk difference curves for the prevention of therapeutic duplication or drug interaction from scoring system I and II with stepwise selection.

**eTable 1**. **The sensitivity analysis of additive interaction for the adjustment of leftover drugs (including individuals who switched groups).**

| **Variables** | **Levels** | **Non-user** | **User** | **aARDs of product terms (95% CIs)** |
| --- | --- | --- | --- | --- |
| Age | < 60 years | REF | 2.1 | -1.5 (-4.8 to 1.8) |
|  | ≥ 60 years | 1.1 | 1.7 |  |
| Sex | Male | REF | 2.4 | -1.2 (-3.8 to 1.5) |
|  | Female | 0.1 | 1.4 |  |
| Types of drugs | < 6 | REF | 2.0 | -0.3 (-3.1 to 2.6) |
|  | ≥ 6 | 0.3 | 2.0 |  |
| Medical institutions used | < 2 | REF | 2.3 | -0.6 (-3.4 to 2.1) |
|  | ≥ 2 | -0.2 | 1.4 |  |
| Use of multiple departments in a hospital | No | REF | 1.8 | 0.6 (-5.0 to 6.2) |
|  | Yes | 0.9 | 3.4 |  |
| Admission | No | REF | 1.6 | 6.3 (-2.3 to 14.9) |
|  | Yes | 0.3 | 8.2 |  |
| Use of one-dose package for drugs | No | REF | 1.7 | 3.9 (-3.8 to 11.6) |
|  | Yes | 3.1 | 8.7 |  |
| Antihypertensives | No | REF | 1.6 | 1.0 (-2.0 to 3.9) |
|  | Yes | 1.0 | 3.5 |  |
| Antilipidemic agents | No | REF | 2.4 | -2.0 (-5.2 to 1.2) |
|  | Yes | 1.2 | 1.6 |  |
| Antidiabetic agents other than insulin | No | REF | 1.9 | -0.7 (-5.8 to 4.4) |
|  | Yes | 3.8 | 5.1 |  |
| Insulin | No | REF | 1.7 | 14.2 (-1.6 to 30.1) |
|  | Yes | 1.6 | 17.5 |  |
| Anticoagulants | No | REF | 1.9 | -0.5 (-9.9 to 8.9) |
|  | Yes | 2.1 | 3.5 |  |
| Antiplatelet agents | No | REF | 1.9 | -2.0 (-8.8 to 4.7) |
|  | Yes | 1.7 | 1.6 |  |
| Proton pump inhibitors | No | REF | 1.7 | 1.7 (-2.5 to 5.8) |
|  | Yes | 1.2 | 4.5 |  |
| H2 blockers | No | REF | 2.0 | -1.7 (-6.5 to 3.0) |
|  | Yes | 0.3 | 0.6 |  |
| H1 blockers | No | REF | 1.7 | 0.5 (-2.3 to 3.3) |
|  | Yes | 0.1 | 2.2 |  |
| Antipsychotics | No | REF | 1.6 | 3.5 (-1.9 to 8.9) |
|  | Yes | 0.8 | 5.9 |  |
| BZDs/Non-BZDs | No | REF | 1.4 | 2.3 (-1.2 to 5.8) |
|  | Yes | 0.5 | 4.2 |  |
| Antidepressants | No | REF | 1.3 | 5.1 (-0.01 to 10.3) |
|  | Yes | 0.5 | 7.0 |  |
| Antibiotics | No | REF | 2.1 | -0.5 (-3.3 to 2.2) |
|  | Yes | -0.1 | 1.5 |  |
| NSAIDs | No | REF | 2.5 | -1.8 (-4.5 to 0.9) |
|  | Yes | 0.1 | 0.8 |  |
| Steroids | No | REF | 2.0 | -1.0 (-5.2 to 3.2) |
|  | Yes | 0.4 | 1.3 |  |
| Probiotics | No | REF | 1.8 | 0.5 (-3.3 to 4.4) |
|  | Yes | 0.1 | 2.4 |  |
| Laxatives | No | REF | 1.9 | 0.2 (-5.7 to 6.2) |
|  | Yes | 0.4 | 2.5 |  |
| Vitamins | No | REF | 1.9 | 0.0 (-4.4 to 4.4) |
|  | Yes | 0.3 | 2.1 |  |
| Traditional Japanese herbal medicines | No | REF | 2.0 | -0.4 (-3.6 to 2.7) |
|  | Yes | 0.3 | 1.8 |  |

Abbreviations. aARDs: adjusted absolute risk differences; BZDs: benzodiazepine; CIs: confidence intervals; NSAIDs: non-steroidal anti-inflammatory drugs; REF: reference.

Value in each stratum indicates aARDs from a common REF stratum.

**eTable 2. The sensitivity analysis of additive interaction for the prevention of therapeutic duplication or drug interaction (including individuals who switched groups).**

| **Variables** | **Levels** | **Non-user** | **User** | **aARDs of product terms (95% CIs)** |
| --- | --- | --- | --- | --- |
| Age | < 60 years | REF | 2.1 | -0.9 (-4.6 to 2.8) |
|  | ≥ 60 years | 0.5 | 1.7 |  |
| Sex | Male | REF | -0.2 | 4.7 (1.6 to 7.9) |
|  | Female | 0.5 | 5.0 |  |
| Types of drugs | < 6 | REF | -1.1 | 4.4 (1.5 to 7.2) |
|  | ≥ 6 | 0.0 | 3.3 |  |
| Medical institutions used | < 2 | REF | -0.9 | 4.6 (1.7 to 7.4) |
|  | ≥ 2 | -0.1 | 3.5 |  |
| Use of multiple departments in a hospital | No | REF | 1.4 | 6.9 (-0.7 to 14.5) |
|  | Yes | 2.2 | 10.5 |  |
| Admission | No | REF | 1.7 | 3.8 (-4.6 to 12.3) |
|  | Yes | 0.6 | 6.1 |  |
| Use of one-dose package for drugs | No | REF | 1.5 | 5.9 (-2.3 to 14.1) |
|  | Yes | 1.8 | 9.2 |  |
| Antihypertensives | No | REF | 1.7 | 0.4 (-3.0 to 3.8) |
|  | Yes | 0.0 | 2.2 |  |
| Antilipidemic agents | No | REF | 2.2 | -1.3 (-4.7 to 2.1) |
|  | Yes | 0.4 | 1.3 |  |
| Antidiabetic agents other than insulin | No | REF | 1.6 | 2.5 (-2.9 to 8.0) |
|  | Yes | 0.6 | 4.7 |  |
| Insulin | No | REF | 1.6 | 14.6 (-1.6 to 30.8) |
|  | Yes | 1.1 | 17.2 |  |
| Anticoagulants | No | REF | 1.7 | 5.9 (-7.2 to 18.9) |
|  | Yes | 0.6 | 8.1 |  |
| Antiplatelet agents | No | REF | 1.9 | 0.3 (-7.9 to 8.5) |
|  | Yes | -0.3 | 1.8 |  |
| Proton pump inhibitors | No | REF | 1.0 | 5.7 (0.5 to 10.8) |
|  | Yes | 1.8 | 8.5 |  |
| H2 blockers | No | REF | 1.8 | 0.6 (-5.9 to 7.1) |
|  | Yes | 1.4 | 3.8 |  |
| H1 blockers | No | REF | 0.9 | 3.1 (-0.6 to 6.7) |
|  | Yes | 0.8 | 4.7 |  |
| Antipsychotics | No | REF | 1.6 | 2.3 (-3.2 to 7.8) |
|  | Yes | 0.3 | 4.2 |  |
| BZDs/Non-BZDs | No | REF | 1.1 | 3.6 (-0.6 to 7.8) |
|  | Yes | 0.6 | 5.2 |  |
| Antidepressants | No | REF | 2.0 | -0.9 (-5.6 to 3.8) |
|  | Yes | 0.0 | 1.1 |  |
| Antibiotics | No | REF | 0.0 | 5.5 (1.9 to 9.0) |
|  | Yes | 0.1 | 5.5 |  |
| NSAIDs | No | REF | 1.5 | 0.9 (-2.4 to 4.3) |
|  | Yes | 0.3 | 2.7 |  |
| Steroids | No | REF | 1.4 | 4.8 (-1.5 to 11.1) |
|  | Yes | 0.8 | 7.0 |  |
| Probiotics | No | REF | 0.8 | 7.9 (2.4 to 13.5) |
|  | Yes | 0.4 | 9.1 |  |
| Laxatives | No | REF | 1.7 | 3.5 (-4.3 to 11.3) |
|  | Yes | 0.5 | 5.6 |  |
| Vitamins | No | REF | 1.7 | 2.2 (-3.7 to 8.2) |
|  | Yes | 0.8 | 4.7 |  |
| Traditional Japanese herbal medicines | No | REF | 0.5 | 6.9 (2.3 to 11.5) |
|  | Yes | 0.5 | 7.9 |  |

Abbreviations. aARDs: adjusted absolute risk differences; BZDs: benzodiazepine; CIs: confidence intervals; NSAIDs: non-steroidal anti-inflammatory drugs; REF: reference.

Value in each stratum indicates aARDs from a common REF stratum.

**eTable 3. The coefficients of multiple logistic regression models for the scoring system I and II for the prevention of therapeutic duplication or drug interaction.**

| **Variables** | **Scoring system I** | | **Scoring system II** |
| --- | --- | --- | --- |
|  | **Coefficients (95% CIs)**  **from user** | **Coefficients (95% CIs)**  **from non-user** | **Coefficients (95% CIs)** |
| User/Non-user | NA | NA | -0.13 (-0.92 to 0.66) |
| Sex | 0.79 (0.24 to 1.33) | 0.21 (0.14 to 0.28) | 0.21 (0.14 to 0.28) |
| Types of drugs | 0.45 (-0.46 to 1.37) | 0.29 (0.19 to 0.40) | 0.30 (0.20 to 0.40) |
| Medical institutions used | 0.14 (-0.58 to 0.85) | 0.05 (-0.03 to 0.14) | 0.06 (-0.03 to 0.15) |
| Proton pump inhibitors | 0.51 (-0.15 to 1.17) | 0.43 (0.34 to 0.52) | 0.43 (0.34 to 0.52) |
| Antibiotics | -0.01 (-0.59 to 0.57) | -0.02 (-0.09 to 0.05) | -0.02 (-0.09 to 0.06) |
| Probiotics | 0.42 (-0.21 to 1.05) | 0.10 (0.01 to 0.19) | 0.10 (0.01 to 0.19) |
| Traditional Japanese herbal medicines | 0.54 (-0.02 to 1.10) | 0.17 (0.09 to 0.25) | 0.17 (0.09 to 0.25) |
| Product terms |  |  |  |
| User/Non-user * Sex | NA | NA | 0.49 (-0.04 to 1.02) |
| User/Non-user * Types of drugs | NA | NA | -0.05 (-0.94 to 0.83) |
| User/Non-user * Medical institutions used | NA | NA | -0.07 (-0.76 to 0.62) |
| User/Non-user * Proton pump inhibitors | NA | NA | -0.17 (-0.77 to 0.42) |
| User/Non-user * Antibiotics | NA | NA | -0.03 (-0.58 to 0.53) |
| User/Non-user * Probiotics | NA | NA | 0.29 (-0.32 to 0.90) |
| User/Non-user * Traditional Japanese herbal medicines | NA | NA | 0.45 (-0.09 to 1.00) |
| Other variables |  |  |  |
| Age | -0.18 (-0.87 to 0.52) | 0.20 (0.11 to 0.29) | 0.19 (0.11 to 0.28) |
| Use of multiple departments in a hospital | 0.56 (-0.24 to 1.37) | 0.43 (0.30 to 0.56) | 0.43 (0.30 to 0.55) |
| Admission | -0.38 (-1.45 to 0.69) | 0.19 (0.04 to 0.34) | 0.18 (0.03 to 0.33) |
| Use of one-dose package for drugs | 0.14 (-0.76 to 1.03) | 0.14 (-0.06 to 0.35) | 0.15 (-0.05 to 0.34) |
| Antihypertensives | 0.06 (-0.53 to 0.66) | 0.11 (0.03 to 0.20) | 0.11 (0.03 to 0.20) |
| Antilipidemic agents | 0.30 (-0.32 to 0.93) | 0.18 (0.09 to 0.27) | 0.18 (0.09 to 0.27) |
| Antidiabetic agents other than insulin | -0.10 (-1.01 to 0.80) | 0.18 (0.05 to 0.30) | 0.17 (0.05 to 0.29) |
| Insulin | 1.34 (-0.11 to 2.78) | 0.33 (0.09 to 0.57) | 0.35 (0.11 to 0.58) |
| Anticoagulants | 0.71 (-0.73 to 2.15) | 0.22 (-0.04 to 0.48) | 0.22 (-0.04 to 0.47) |
| Antiplatelet agents | 0.14 (-0.97 to 1.24) | 0.00 (-0.18 to 0.18) | 0.00 (-0.17 to 0.18) |
| H2 blockers | 0.23 (-0.58 to 1.04) | 0.35 (0.24 to 0.45) | 0.35 (0.24 to 0.45) |
| H1 blockers | 0.76 (0.20 to 1.32) | 0.28 (0.21 to 0.35) | 0.28 (0.21 to 0.35) |
| Antipsychotics | 0.22 (-0.63 to 1.08) | 0.24 (0.10 to 0.38) | 0.24 (0.09 to 0.38) |
| BZDs/Non-BZDs | 0.57 (-0.10 to 1.24) | 0.29 (0.19 to 0.38) | 0.29 (0.20 to 0.39) |
| Antidepressants | -0.35 (-1.19 to 0.49) | 0.06 (-0.07 to 0.19) | 0.06 (-0.07 to 0.19) |
| NSAIDs | -0.56 (-1.13 to 0.01) | 0.16 (0.08 to 0.23) | 0.14 (0.07 to 0.21) |
| Steroids | -0.27 (-1.06 to 0.51) | 0.24 (0.14 to 0.34) | 0.23 (0.13 to 0.33) |
| Laxatives | -0.47 (-1.47 to 0.54) | 0.05 (-0.09 to 0.18) | 0.03 (-0.10 to 0.16) |
| Vitamins | 0.31 (-0.38 to 1.01) | 0.23 (0.14 to 0.33) | 0.23 (0.14 to 0.33) |
| The number of medical examinations | 0.01 (-0.01 to 0.03) | 0.02 (0.02 to 0.02) | 0.02 (0.02 to 0.02) |
| Use of a pharmacy that can claim the family pharmacist consultation fees | NA | 0.56 (0.49 to 0.63) | 0.56 (0.49 to 0.63) |

Abbreviations. BZDs: benzodiazepine; CIs: confidence intervals; NA, not applicable; NSAIDs: non-steroidal anti-inflammatory drugs.

**eTable 4. The coefficients of multiple logistic regression models for the scoring system I and II for the prevention of therapeutic duplication or drug interaction with stepwise selection.**

| **Variables** | **Scoring system I** | | **Scoring system II** |
| --- | --- | --- | --- |
|  | **Coefficients (95% CIs)**  **from user** | **Coefficients (95% CIs)**  **from non-user** | **Coefficients (95% CIs)** |
| User/Non-user | NA | NA | -0.22 (-0.67 to 0.23) |
| Sex | 0.80 (0.26 to 1.35) | 0.21 (0.14 to 0.28) | 0.21 (0.14 to 0.28) |
| Types of drugs | excluded | 0.31 (0.22 to 0.40) | 0.30 (0.20 to 0.40) |
| Medical institutions used | excluded | excluded | 0.06 (-0.03 to 0.15) |
| Proton pump inhibitors | 0.57 (-0.09 to 1.23) | 0.43 (0.34 to 0.52) | 0.43 (0.34 to 0.52) |
| Antibiotics | excluded | excluded | -0.02 (-0.09 to 0.05) |
| Probiotics | 0.46 (-0.15 to 1.08) | 0.09 (0.01 to 0.18) | 0.10 (0.01 to 0.19) |
| Traditional Japanese herbal medicines | 0.62 (0.08 to 1.17) | 0.17 (0.09 to 0.25) | 0.17 (0.09 to 0.25) |
| Product terms |  |  |  |
| User/Non-user * Sex | NA | NA | 0.48 (-0.04 to 1.01) |
| User/Non-user * Types of drugs | NA | NA | excluded |
| User/Non-user * Medical institutions used | NA | NA | excluded |
| User/Non-user * Proton pump inhibitors | NA | NA | excluded |
| User/Non-user * Antibiotics | NA | NA | excluded |
| User/Non-user * Probiotics | NA | NA | excluded |
| User/Non-user * Traditional Japanese herbal medicines | NA | NA | 0.47 (-0.06 to 0.99) |
| Other variables |  |  |  |
| Age | -0.20 (-0.89 to 0.50) | 0.20 (0.12 to 0.29) | 0.20 (0.11 to 0.28) |
| Use of multiple departments in a hospital | 0.57 (-0.24 to 1.37) | 0.43 (0.30 to 0.55) | 0.43 (0.30 to 0.55) |
| Admission | -0.35 (-1.42 to 0.72) | 0.19 (0.04 to 0.35) | 0.18 (0.02 to 0.33) |
| Use of one-dose package for drugs | 0.14 (-0.75 to 1.04) | 0.14 (-0.06 to 0.34) | 0.15 (-0.05 to 0.34) |
| Antihypertensives | 0.07 (-0.52 to 0.66) | 0.11 (0.03 to 0.20) | 0.11 (0.03 to 0.20) |
| Antilipidemic agents | 0.31 (-0.31 to 0.93) | 0.18 (0.09 to 0.27) | 0.18 (0.09 to 0.27) |
| Antidiabetic agents other than insulin | -0.09 (-1.00 to 0.81) | 0.17 (0.05 to 0.30) | 0.17 (0.05 to 0.29) |
| Insulin | 1.35 (-0.09 to 2.80) | 0.33 (0.09 to 0.57) | 0.35 (0.12 to 0.59) |
| Anticoagulants | 0.65 (-0.81 to 2.12) | 0.22 (-0.04 to 0.48) | 0.22 (-0.04 to 0.47) |
| Antiplatelet agents | 0.16 (-0.95 to 1.27) | 0.00 (-0.18 to 0.18) | 0.00 (-0.17 to 0.18) |
| H2 blockers | 0.27 (-0.54 to 1.08) | 0.35 (0.24 to 0.46) | 0.35 (0.24 to 0.45) |
| H1 blockers | 0.84 (0.29 to 1.38) | 0.28 (0.21 to 0.34) | 0.28 (0.21 to 0.35) |
| Antipsychotics | 0.22 (-0.63 to 1.08) | 0.24 (0.10 to 0.39) | 0.24 (0.09 to 0.38) |
| BZDs/Non-BZDs | 0.59 (-0.08 to 1.27) | 0.29 (0.19 to 0.39) | 0.29 (0.20 to 0.39) |
| Antidepressants | -0.32 (-1.16 to 0.53) | 0.06 (-0.07 to 0.20) | 0.06 (-0.07 to 0.18) |
| NSAIDs | -0.48 (-1.03 to 0.07) | 0.16 (0.09 to 0.23) | 0.14 (0.07 to 0.21) |
| Steroids | -0.26 (-1.04 to 0.53) | 0.24 (0.14 to 0.34) | 0.23 (0.13 to 0.33) |
| Laxatives | -0.46 (-1.48 to 0.55) | 0.05 (-0.09 to 0.18) | 0.03 (-0.10 to 0.17) |
| Vitamins | 0.32 (-0.37 to 1.02) | 0.23 (0.14 to 0.33) | 0.23 (0.14 to 0.33) |
| The number of medical examinations | 0.01 (-0.01 to 0.03) | 0.02 (0.02 to 0.02) | 0.02 (0.02 to 0.02) |
| Use of a pharmacy that can claim the family pharmacist consultation fees | NA | 0.56 (0.49 to 0.63) | 0.56 (0.49 to 0.63) |

Abbreviations*.* BZDs: benzodiazepine; CIs: confidence intervals; NA, not applicable; NSAIDs: non-steroidal anti-inflammatory drugs.

**eTable 5. Score allocations of the identified modifiers for the prevention of therapeutic duplication or drug interaction from scoring system I and II with stepwise selection.**

| **Variables** | **Scoring system I with stepwise selection** | **Scoring system II with stepwise selection** |
| --- | --- | --- |
| Sex (Female) | 0.59 | 0.48 |
| Types of drugs (≥ 6) | -0.31 | 0.00 |
| Medical institutions used (≥ 2) | 0.00 | 0.00 |
| Proton pump inhibitors | 0.15 | 0.00 |
| Antibiotics | 0.00 | 0.00 |
| Probiotics | 0.37 | 0.00 |
| Traditional Japanese herbal medicines | 0.45 | 0.47 |

**eTable 6. Correspondence between scores and aARDs for the prevention of therapeutic duplication or drug interaction from scoring system I and II with stepwise selection.**

|  | **Scoring system I**  **with stepwise selection** | | | | **Scoring system II**  **with stepwise selection** | | | |
| --- | --- | --- | --- | --- | --- | --- | --- | --- |
|  | **Training** | | **Test** | | **Training** | | **Test** | |
| **q** | **Scores** | **aARDs** | **Scores** | **aARDs** | **Scores** | **aARDs** | **Scores** | **aARDs** |
| 0 | -0.31 | 1.4 | -0.31 | 3.5 | 0.00 | 1.4 | 0.00 | 3.5 |
| 10 | -0.31 | 1.4 | -0.31 | 3.5 | 0.00 | 1.4 | 0.00 | 3.5 |
| 20 | -0.16 | 1.5 | -0.16 | 4.3 | 0.00 | 1.4 | 0.00 | 3.5 |
| 30 | 0.00 | 1.8 | 0.00 | 3.9 | 0.00 | 1.4 | 0.00 | 3.5 |
| 40 | 0.00 | 1.8 | 0.06 | 4.8 | 0.00 | 1.4 | 0.00 | 3.5 |
| 50 | 0.28 | 3.4 | 0.28 | 5.4 | 0.47 | 3.1 | 0.47 | 5.1 |
| 60 | 0.28 | 3.4 | 0.28 | 5.4 | 0.48 | 3.7 | 0.48 | 5.8 |
| 70 | 0.51 | 6.1 | 0.51 | 6.6 | 0.48 | 3.7 | 0.48 | 5.8 |
| 80 | 0.59 | 6.0 | 0.59 | 7.0 | 0.48 | 3.7 | 0.48 | 5.8 |
| 90 | 0.73 | 9.3 | 0.73 | 11.7 | 0.95 | 11.6 | 0.95 | 9.5 |
| 100 | 1.56 | 12.8 | 1.56 | 64.2 | 0.95 | 11.6 | 0.95 | 9.5 |
| AUCs | 483.3 | | 744.8 | | 359.4 | | 518.1 | |

Abbreviations. aARDs: adjusted absolute risk differences; AUCs: the areas under the adjusted absolute risk difference curves.

**eFigure 1. Adjusted absolute risk difference curves for the prevention of therapeutic duplication or drug interaction from scoring system I and II with stepwise selection.**


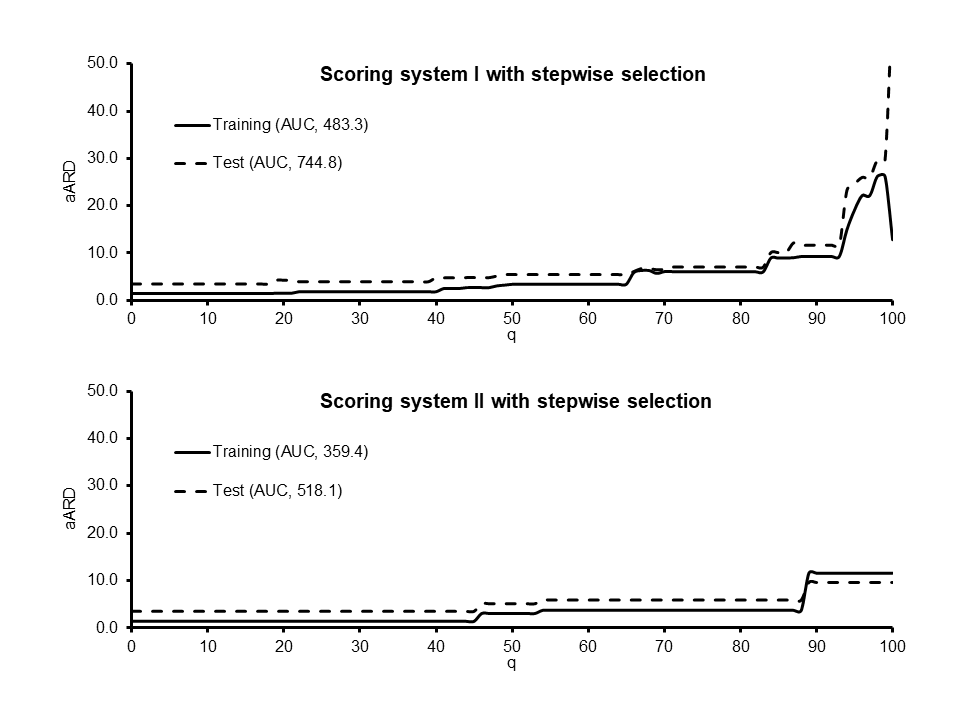


Abbreviations*.* aARD: adjusted absolute risk difference; AUC: area under the absolute risk difference curve.

Scoring system I was developed using two multiple logistic regression models with stepwise selection and scoring system II was developed using a single multiple logistic regression model with stepwise selection. The plots depict q in the x-axis and the corresponding aARDs in the y-axis based on these scoring systems. The higher the AUC is, the better the scoring system performs.
